# The Impact of Digital Healthcare Adoption and Service Quality on Patient Satisfaction: The Moderating Role of Telehealth Services in Pakistan

**DOI:** 10.1155/jonm/8184283

**Published:** 2026-06-28

**Authors:** Shahida Kanwel, Zhiqiang Ma, Arif Jameel, Mingxing Li, Abid Hussain, Bailin Ge, Bahar Hussain, Saif Ahmed

**Affiliations:** ^1^ School of Management, Jiangsu University, Zhenjiang, 212013, Jiangsu, China, ujs.edu.cn; ^2^ School of Business, Shandong Xiehe University, Jinan, 250109, China, sdxiehe.edu.cn; ^3^ School of Nursing, Shandong Xiehe University, Jinan, 250109, China, sdxiehe.edu.cn; ^4^ Institute of Business Management Sciences, University of Agriculture, Faisalabad, 03802, Pakistan, uam.edu.ng; ^5^ Department of Public Administration, The Islamia University, Bahawalpur, 63100, Punjab, Pakistan, iub.edu.pk

**Keywords:** digital healthcare adoption, doctor–patient communication, patient satisfaction, telehealth services

## Abstract

Digital services allow patients to efficiently access healthcare. These services work more effectively than traditional paper‐based systems by delivering better patient outcomes, helping address global health challenges, and promoting the universal adoption of health technology. This study examined the impact of digital healthcare adoption and service quality on patient satisfaction in Pakistan’s public healthcare sector and the moderating effect of telehealth services on this relationship. This study adopted the technology acceptance model to understand technology sophistication and how electronic medical records, digital patient systems, and technology impact healthcare through efficiency and communication. Simultaneously, the study examined the role of doctor services, nurse services, pharmacy services, and laboratory services in the patient experience. Random sampling techniques were employed, and questionnaires were distributed to 573 respondents across five central districts of Punjab, Pakistan. The hypotheses were tested using IBM SPSS Statistics, Amos, and structural equation modeling. These findings show that digital healthcare adoption and service quality significantly improve patient satisfaction, whereas telehealth services reinforce these relationships by overcoming geographical and logistical hurdles. The conclusions of this study offer pragmatic guidance to policymakers and healthcare administrators for devising digital healthcare strategies to improve patient outcomes.

## 1. Introduction

Digital technologies have rapidly advanced medical service delivery systems across all healthcare systems worldwide. Healthcare systems in developed countries have transitioned patient data from paper to digital formats by utilizing electronic health records (EHRs), digital patient record (DPR) platforms, and telehealth services (TSs) to better serve their patients [1]. Decades ago, the United States, Germany, and the United Kingdom introduced digital tools into healthcare to simplify doctors’ messages and prevent errors, ultimately making medical care more accessible to patients [2], while developing countries such as China, Malaysia, and Singapore have invested in digital solutions utilizing mobile health (mHealth) and telehealth to provide healthcare to their populations.

Information technology has generally received global acceptance for the delivery of healthcare; however, its applicability and success rates differ in various parts of the world, especially in developing countries [3]. The lack of equity in meeting societal needs, inequalities in human ability to use technology, and lack of infrastructure to implement a fully paperless or computer‐based health system are some of the issues affecting a fully digitalized healthcare system. Although digital healthcare is still a nascent issue in most developing settings, the situation in Pakistan, with its under‐resourced governmental facilities, system of physical cash payment in rural healthcare, and lack of technological preparedness, makes it stand out from the rest of the world in terms of digital health transformation [4].

Pakistan’s public health system has a weak infrastructure, inadequately equipped public hospitals, and an expensive private healthcare system. These challenges include a lack of adequate health facilities, a shortage of healthcare workers, poor record‐keeping systems, and inadequate healthcare policies and access to medical care, especially in rural areas. These challenges have prompted the government and private sector to adopt and implement digital solutions in healthcare services [5]. An assessment of the impact of introducing EHRs in private hospitals revealed encouraging signs of patient management. However, most public health institutions have not fully adopted them due to policies and regulations, financial constraints, and technological hurdles [6].

The low adoption of mobile money in rural and underserved regions poses a structural challenge to the digital healthcare transformation. Digital health services such as teleconsultations, EHR‐driven follow‐up payments, electronic prescription refills, and online booking of diagnostic tests are not possible without integrated digital payment systems (DPSs) to complete the service payment lifecycle [7, 8]. In healthcare facilities that use cash‐based systems as the primary solution, patients cannot complete digital workflows, which disrupts the continuity of care, as well as the usability of telehealth and EMR‐based services [9]. Moreover, the lack of secure digital channels of payment restricts providers’ capacity to charge for remote care and prevents the growth of digital care models in resource‐limited areas [10]. This leads to a reduced uptake of mobile money as a bottleneck that limits digital health uptake, especially in rural Pakistan, where infrastructural and financial digitalization is poor [5, 11].

Digital health technology is evolving and is an essential component for achieving the extent of technological advancement [12]. At the same time, infrastructural support limitations, such as erratic Internet connections or the lack of a proper cybersecurity plan, impede its advancement. This study established that service quality is one of the most important antecedents of patient satisfaction (PS) in Pakistan’s healthcare industry. This implies that the quality of services provided by doctors, the care offered by nurses, the availability and quality of the pharmacy, and the performance of the laboratory significantly shaped patients’ experiences. However, there are disparities in service delivery between public and private health facilities as well as in the level of information and computer technology (ICT) utilization in healthcare service provision.

**FIGURE 1:**
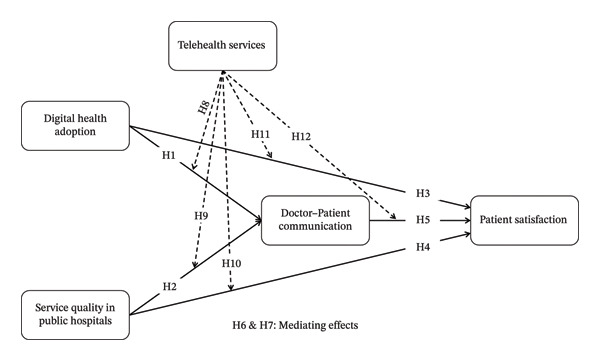
Proposed framework. H6 and H7: Mediating effects.

This study contributes to the literature by incorporating digital healthcare adoption (DHA) and service quality dimensions to evaluate their combined effects on PS in Pakistan’s public health sector. This study situates Pakistan within the global context of digitalization, facilitating comparisons that will inform policymakers, healthcare decision‐makers, and technology designers. The findings provide insights into how these concepts and service quality factors relate to the best practices of digital healthcare and the specific issues that a country’s developing health system presents. Refer to Figure 1 for the proposed study model.

## 2. Study Novelty and Contribution to the Literature

### 2.1. Contextual Novelty

The current state of public hospitals in Pakistan, particularly hospitals in central Punjab, is associated with low digital maturity, dispersed use of EHRs, weak ICT infrastructure, and disproportionate telehealth implementation. Such systemic limitations make an acute contrast with the level of digital preparedness of countries in which the technology acceptance model (TAM) has been thoroughly tested, including the United States, Europe, and East Asia [1, 2, 13]. Digital illiteracy, infrastructural insufficiency, and policy fragmentation are some of the most common challenges observed in digital health research in low‐ and medium‐income countries (LMICs) [4, 8, 12], and the number of studies conducted on the TAM in the context of hospitals of the Pakistani public sector is virtually zero. By placing the TAM in a resource‐bound, policy‐divided system of public health, this study reveals adoption mechanisms that are markedly different in digitally mature settings. This contextual gap makes this study an important continuation of DHA research in low‐resource public health settings.

### 2.2. Model Novelty

This research is an extension of the TAM, wherein four fundamental service quality services, namely, doctor services, nurse services, pharmacy services, and laboratory services, are incorporated into digital technology adoption for PS. Previous digital health studies have usually measured these constructs independently [14–16]. Nevertheless, only a few studies have integrated them within a single TAM‐based structural framework in publicly controlled healthcare settings. This integration enabled us to analyze the behavioral interaction of technology acceptance and service delivery processes to provide a more detailed behavioral model of the digital health experience.

### 2.3. Moderation Novelty

The present study is the first empirical study to be conducted in Pakistan to assess the multipath moderating effect of TSs while simultaneously moderating the effect. The existing telehealth literature in Pakistan and similar LMICs generally focuses on the direct impact on satisfaction with or access to telehealth [17–19] and does not investigate telehealth as a mediating factor that intensifies or changes the pathways across various structural arenas. We introduce a new lens of analysis on how telehealth enhances or limits technology‐mediated patient outcomes, a dimension that has been understudied in DHA studies. These contextual, model‐based, and moderation‐centered innovations enhance the novelty of this study and add new theoretical knowledge to the overloaded TAM and digital health body of work. The results expand TAM beyond mere acceptance behaviors by showing that the interaction between digital adoption, service quality, and telehealth infrastructure reflects PS within an underdigitalized population healthcare context.

### 2.4. Research Objectives and Structure

This study sets the following main research goals:

1. This study examined the impact of DHA on PS in public healthcare institutions across Pakistan.
2. This study examined the impact of healthcare service quality—specifically, doctor services, nurse services, pharmacy services, and laboratory services—on PS.
3. This study investigated the impact of telehealth on the relationships among DHA, service quality, and PS.

### 2.5. TAM Core Variables

TAM is one of the most well‐known theories used to describe or explain the user acceptance of technology. Initially proposed by Davis [20], the TAM consists of two variables: perceived usefulness (PU) and perceived ease of use (PEOU). PU is one of the most essential constructs that deal with the specific beliefs a user has concerning the impact of technology on their work. PEOU is another factor related to the amount of effort a person must exert to use a particular technology [21]. These factors are the determinants of behavioral intention and consistently influence the use of a system in real‐life situations. To date, the TAM has been applied in different fields as a tool to predict and explain attitudes toward accepting new technological solutions, including the healthcare sphere [12, 13].

### 2.6. Evolution of TAM and Its Relevance to Digital Healthcare

Since its 1989 release, the TAM has been refined to a point that has allowed it to increase its explanatory ability in a wide range of technological and service settings. Although the original conceptualization of the TAM constituted the adoption of technology in relation to PU and PEOU, later research expanded the concept to better represent behavioral, social, and contextual factors that affect the adoption of technology, especially in complicated service settings such as healthcare.

Venkatesh and Davis [22] developed TAM2, which added social influence mechanisms (e.g., subjective norms and image) and cognitive instrumental mechanisms (e.g., output quality and job relevance) to the original model. These extensions enhance the explanatory power of TAM, particularly in organizational contexts where the use of technology can be due to peer pressure, managerial support, or even perceived professional usefulness. Subsequently, the model was further developed by TAM3 [23] to identify a wide range of determinants of PEOU, including computer self‐efficacy, external control perceptions, and computer anxiety. As the adoption of digital tools in healthcare facilities has frequently been considered a “strange” concept, on both the part of the provider and the patient, TAM3 provides a perspective that is applicable to the current issue by recognizing the psychological and experience‐related barriers that affect technology acceptance.

In 2003, the unified theory of acceptance and use of technology (UTAUT) by Venkatesh et al. consolidated eight competing models of technology adoption, including TAM, to formulate a more integrated explanatory model. UTAUT proposed performance expectancy, effort expectancy, facilitating conditions, and social influence as the main predictors of technology usage intentions and behavior. Hedonic motivation, price value, and habit were later integrated into UTAUT2, rendering the model more consumer‐appropriate [24]. These UTAUT extensions are particularly relevant to the informational context of developing countries, where infrastructural limitations and user willingness play important roles in adopting digital health technologies.

Regarding digital healthcare, other determinants (not limited to PU and PEOU) should be included in adoption behavior. The factors of trust in technology, privacy, and security and facilitating factors are especially topical in the field of healthcare because the utilization of electronic medical data, telehealth, artificial intelligence (AI), and DPS touch on personal sensitive data, ethical vulnerability, and a greater perceived risk. Thus, while the TAM offers a background for the concept of technology acceptance in the healthcare setting, an extended TAM is required to support the role played by service experiences, perceived service quality, and satisfaction with digital interaction, which ultimately affect patient outcomes.

Considering this development, the present study builds on the TAM and incorporates three fundamental constructs into the digital healthcare system: service quality, DHA, and TSs. This method moves the TAM to a point of intention‐to‐use for actual PS, which makes the procedure more in line with social healthcare performance goals. The addition of service quality acknowledges that, even with the use of technology, PS cannot be improved unless such adoption is supported by quality healthcare provisions. Moreover, telehealth was included as a moderating mechanism, illustrating the increased role of this technology in improving care continuity, accessibility, and patient participation. This continuation is consistent with the modern demands for patient‐focused modifications of the TAM, especially in developing nations, where digitalization intersects with infrastructural, social, and quality‐of‐care issues.

### 2.7. Extending TAM for DHA and PS

Since healthcare service delivery is vital, service quality can be considered one of the determinants of PS within the scope of the TAM extensions in this research. A common understanding of service quality in healthcare is based on reliability, responsiveness, assurance, and tangible dimensions as the determinants of patient perceptions and experiences [13]. Finally, in keeping with the surroundings of this study, the core public healthcare services were further operationalized by the quality of service of doctors, nurses, pharmacies, and laboratory services, which are more indicative of patient experience in public hospitals.

In the case of DHA, three additional factors are considered beyond PU and PEOU: trust in technology, privacy and security concerns, and facilitating conditions [12]. The factors that affect patients’ and healthcare providers’ trust in technology and, hence, their willingness to adopt digital health platforms are trust in technology, privacy, and security concerns, where the latter affects their willingness to provide personal health data on platforms if they do not believe that their data would be safe. Facilitating conditions such as infrastructure and technical support are determinants that ease adoption and utilization [25].

Patients must feel satisfied with digital healthcare and its achievement of excellent service results, as patients’ perceptions of digital healthcare services determine how effectively they utilize them. When patients perceive their healthcare experience as helpful and easy and receive high‐quality services, they tend to express greater satisfaction. When patients trust that their data remain safe, they become more satisfied with their healthcare experiences [26].

### 2.8. DHA and Doctor–Patient Communication

The digital adoption of healthcare services such as EHRs, DPS, and advanced technology enhances communication between doctors and patients by providing improved access to accurate information and more effective digital chat systems [27]. EHRs enable doctors and patients to connect efficiently by enabling them to view the latest health records and reports in real time. DPS simplifies financial procedures in healthcare facilities to help healthcare professionals spend less time on payment while doing more patient work [16]. Technology sophistication refers to the level of development and implementation of digital technologies in healthcare institutions, extending beyond basic systems, such as EHRs and decision‐support systems. Technology sophistication serves as an indicator of how extensively hospitals adopt advanced digital infrastructure, such as telemedicine, AI‐driven clinical decision support, remote patient monitoring, and mobile health applications for patients. These technologies have enhanced the functionality, efficiency, and accessibility of healthcare services. In accordance with Andrews et al. [28], technology sophistication is conceptualized as a capability‐based construct that reflects the digital maturity of healthcare organizations, rather than focusing on specific healthcare services. Digital innovations in DHA create better systems for efficient healthcare communication between patients and providers, enhancing patient care results and satisfaction [29].

Although digital innovations can enhance communication effectiveness and patient–provider interactions, not all of their consequences are favorable. The adoption of digital tools in numerous healthcare systems, particularly in developing environments, has had both beneficial and adverse outcomes because of obstacles such as low digital literacy, insufficient infrastructure, user resistance, and workflow disruptions when undergoing digital transformation [3, 8]. Therefore, the success of DHA in improving doctor–patient communication strongly relies on the readiness of the context and effective implementation in clinical processes, but not on the implementation of technologies only. This study proposes the following hypothesis:

 H1: DHA positively impacts doctor–patient communication.

### 2.9. Service Quality and Doctor–Patient Communication

Service quality in public health improves doctor–patient communication through a heightened healthcare setting that provides better administration and prompt service, ultimately leading to patient‐centric care. Doctor services provide transparent and effective communication pathways through precise diagnoses, counseling sessions, and decision‐sharing opportunities that help patients fully understand their medical conditions and treatment strategies [16]. The essential role of nurse services in healing communication stems from their commitment to providing ongoing assistance and education on medications and procedures, with sincere attention paid to responding to patient concerns. Patient communication improves from pharmacy services through explicit information about medications, benefits, and risk management strategies, thus minimizing missed instructions and treatment‐related complications [30]. Laboratory services assist medical staff in their communication efforts through prompt and accurate diagnostics, allowing doctors to make wise decisions before presenting results to patients [31]. The combination of these service quality dimensions enhances trust and clarity while boosting patient engagement during doctor–patient interactions, ultimately yielding better healthcare outcomes. Thus, this study proposes the following hypothesis:

 H2: Service quality positively impacts doctor–patient communication.

### 2.10. DHA and PS

DHA helps patients obtain better care by making services faster and easier to use. EHRs help healthcare providers share patient information more effectively and reduce the likelihood of errors in medical practices. Patients benefit from better treatment results and faster decisions made at healthcare facilities, which increase their trust in healthcare services [32]. DPS technology enables patients to make secure payments through digital channels without any issues. DPS connect healthcare services better by making them faster and simpler to handle, which improves patients’ feelings about their medical care [16].

Healthcare technology, including advanced diagnostic tools, telemedicine, and AI‐driven decision‐support systems, helps both health staff and patients build stronger trust and engagement in care services [31]. High‐tech innovations enhance the accuracy of medical therapy and patient control in treatment plans. Through TSs that expand the DHA framework, patients can remotely connect with healthcare providers to access continuous health monitoring and deliver high‐quality care across all regions, including remote areas. DHA through EMR integration alongside DPS with technological modernization establishes patient‐focused care and builds healthcare consumer satisfaction with service quality [1, 33]. Thus, this study proposes the following hypothesis:

 H3: DHA positively influences PS.

### 2.11. Service Quality in Public Health

Service quality affects PS because it describes how well medical centers perform their duties. Doctor services represent a significant part of service quality by affecting how patients feel about their medical treatment [1]. Patients’ trust in their doctors depends on their specialist knowledge and professionalism, as well as their ability to interact effectively with patients. Better health outcomes and happier patients align with superior diagnostic service quality through precise medical judgment and prompt personal attention provided by healthcare providers. Patients feel better about healthcare when their doctor involves them in decisions and talks to them in a person‐centered manner [34].

Nurse services play a central role in delivering PS by providing ongoing and compassionate care. The primary healthcare responsibilities of nurses include providing primary medical care, offering emotional support, administering treatment, and monitoring patient status. Nurse services quality depends primarily on the responsiveness and empathy of nursing staff members who pay attention to clients, resulting in patients developing perceptions of care quality. The implementation of efficient nurse services improves patient comfort and recovery and decreases anxiety and stress levels, especially when treating large numbers of patients in public healthcare facilities [35]. Professional nursing practice combined with expert performance by nurses increases patients’ trust in healthcare institutions, thereby improving their overall PS.

Pharmacy services play a crucial role in ensuring the availability, accessibility, and accuracy of prescribed medications, thereby contributing to PS. Pharmacy services are efficient in reducing the risk of medication errors, ensuring timely drug dispensing, and providing clear instructions for medication use to patients. Pharmacy services that are well organized and pharmacist–patient interactions focused on guidance and safety increase patient confidence in their treatment plan [36]. Furthermore, they help reduce wait times and maintain cost‐effective access to medicines, facilitating a better overall experience in the healthcare system, which, in turn, makes them an integral part of the quality of public health services.

Laboratory services are essential for PS because they facilitate accurate and timely diagnosis of patients’ conditions to aid doctors in their decision‐making processes [37]. Innovative laboratory services best practices include skilled human resources, advanced technology, and proper sample handling, which help avoid errors in diagnosing illnesses, thereby improving treatment outcomes. This is because delays in laboratory results can affect the time required to diagnose a patient. Subsequently, the time taken before a definite treatment is administered to a particular patient can significantly affect PS. Conversely, functional laboratory services enhance patient trust in medical care because it enables an appropriate and timely diagnosis and, thus, a healthier status [38]. Therefore, it is crucial to implement systems, such as doctor services, nurse services, pharmacy services, and laboratory services, to enhance service quality and increase PS. Thus, this study proposes the following hypotheses:

 H4: Service quality positively influences PS.

Effective doctor–patient communication brings about higher PS because it develops faith, together with comprehension and cooperative bonds [15]. Patients experience greater therapeutic respect when doctors demonstrate clarity in communication and attentive listening, along with emotional understanding. Medical staff who provide detailed information regarding patient diagnoses, treatment choices, and projected outcomes help patients feel confident while reducing their anxiety about medical decisions. Treatment success rates improve when patients feel that their healthcare providers acknowledge their health concerns [19]. A positive relationship between healthcare providers and patients delivers better emotional well‐being outcomes, which, in turn, boosts PS. Healthcare professionals who excel in communication can create environments that support patients’ needs, thereby enhancing their overall medical care experience. Thus, this study proposes the following hypothesis:

 H5: Doctor–patient communication positively influences PS.

### 2.12. Mediating Role of Doctor–Patient Communication Between DHA, Service Quality, and PS

The quality of doctor–patient discussions connects EMR and DPS technology sophistication with PS. EHRs help doctors communicate more effectively with patients because they can access complete medical information, which supports informed discussions and personalized treatment. DPSs streamline office work, enabling doctors to provide better services to patients and focus on patient care rather than administrative tasks [1]. Advanced healthcare technologies enable doctors to connect with and guide patients through telemedicine and AI systems. Digital healthcare tools that enhance communication between doctors and patients foster better treatment trust and patient participation, leading to improved healthcare satisfaction [12].

The quality of doctor–patient interactions serves as a primary linkage mechanism connecting public health service quality aspects, including doctor and nurse services and PS, by building trust and enhancing clarity during healthcare interactions [9]. Quality diagnosis and shared decision‐making abilities of high‐quality doctor services enable physicians to deliver precise medical protocols to patients while building patient‐centered interactions that improve communication effectiveness [26]. Nurse services staff enhance doctor–patient relationships by maintaining continuous medical care, education, and emotional support, which validates doctors’ medical directions [35]. High‐quality service delivery achieved through effective communication enables healthcare providers to connect more effectively with patients, thereby improving their healthcare experience, adherence to treatment plans, and overall satisfaction with public healthcare services. Thus, this study proposes the following hypotheses:

 H6: Doctor–patient communication mediates between DHA and PS.
 H7: Doctor–patient communication mediates between service quality and PS.

### 2.13. The Moderating Role of TSs

Telehealth can act as a manipulation variable between the digitalization of healthcare, service quality, and PS to make services more accessible, efficient, and continuous [39]. As a subdivision of digital healthcare, telehealth is connected to a lack of geographic and logistical solutions that provide the opportunity to receive medical services, even in regions with limited access to such services [16]. When applied to EHRs, telehealth enhances the continuity and timely transfer of patients’ medical records and treatment plans. Likewise, telehealth platforms supporting DPS help secure and convenient financial transactions, eliminate paperwork, and improve PS [17]. Advancements in technology, such as AI diagnosis and virtual consultations, enhance the delivery of healthcare services and avert the delivery of inadequate services, leading to PS [40].

In addition to modifying the effect of service quality dimensions on PS, telehealth helps to create a digital interface for healthcare adoption [15]. Telehealth platforms that support the use of virtual consultations tend to make doctor services more efficient, reduce waiting times, and provide timely medical advice. Remote‐monitoring technology enhances nurse services because it allows nurses to monitor patients’ progress continuously and for a long time, even outside the hospital [40]. For pharmacy services, telehealth allows e‐prescriptions and medication delivery to reach patients as rapidly as possible to access the necessary drugs [41]. Furthermore, the utilization of telehealth by laboratory services provides opportunities for remote consultations of results and helps reduce the visits required to obtain results in person, providing patient convenience and increasing trust on the part of the patient in the healthcare system [39].

Telehealth can be described as an enabler that interoperates health informatics with the adoption of digital healthcare services, thereby enhancing service quality and PS. Telehealth widens patients’ access to healthcare professionals’ expertise, increases the availability of resources through the decentralization of some services, lessens the burden of travel, and improves the efficiency of service delivery in the general population [18, 28]. However, its usefulness is limited by certain conditions, including digital literacy, Internet connectivity, and regulatory environments. The moderating role of telehealth implies that its application enhances the benefits of EHRs, DPS, technology sophistication, and healthcare services in achieving high levels of PS in the public health context.

One of the currently evolving areas within digital healthcare is telehealth, which has been on the rise in recent years, especially in the wake of the COVID‐19 outbreak [28]. Telehealth plays a crucial role in reducing barriers to healthcare access in Pakistan as it provides remote consultations, online prescriptions, and follow‐up care. However, its role in overall PS, which is increasingly linked to digital healthcare and services, remains unclear in qualitative research.

TSs act as moderating variables that enhance the relationships among DHA, service quality, and PS. Telehealth initiatives lower barriers to healthcare delivery, thereby boosting service efficiency, primarily in rural communities that lack adequate medical facilities. Research continues to evaluate the effectiveness of TSs owing to outstanding concerns regarding digital literacy deficiencies, provider reluctance, and patient concerns about virtual care interactions. Thus, this study proposes the following hypotheses:

 H8: TSs moderate between DHA and doctor–patient communication.
 H9: TSs moderate between service quality and doctor–patient communication
 H10: TSs moderate between service quality and PS.
 H11: TSs moderate between DHA and PS.
 H12: TSs moderate between doctor–patient communication and PS.

### 2.14. Conceptual Model

The proposed framework is shown in Figure 1.

## 3. Research Methods

### 3.1. Context Selection

The study used a random sampling technique to adapt questionnaires to patients in public hospitals from August 5, 2024, to December 27, 2024. This data collection method is prevalent in the social sciences and was employed to collect data that would assist in testing the research hypotheses. The study was conducted in five public hospitals across the central districts of Punjab Province, Pakistan, specifically in Lahore, Okara, Nankana Sahib, Sialkot, and Faisalabad, from Monday to Saturday during working hours. The questionnaire consisted of two parts: The first part collected demographic information, and the second part assessed the extent of DHA in terms of EHRs, DPS, and technology sophistication. It also inquired about the dimensions of healthcare service quality, including the services of doctors and nurses, pharmacy services, laboratory and inpatient services, communication with doctors, PS, and TSs. Based on the sampling criteria of Saunders et al. [42], and Krejcie and Morgan [43], the sample size was 750. However, as a final note, we achieved a 76% response rate and collected 573 completed questionnaires.

To achieve representativeness of the sampled hospitals, we selectively selected five large public sector hospitals managed by the unified provincial healthcare governance system in Punjab, which upholds similar operational, regulatory, and digitalization policies. Additionally, a pilot study with 53 subjects was conducted to establish the clarity of the items and the interhospital reliability of the scale. The high level of Cronbach’s alpha is evidence of the consistency of the responses to telehealth constructs in the participants of various locations. This model allows for the comparability of TS experiences in hospitals and enhances confidence in analyzing the moderating effects.

The demographic details of the 573 respondents are presented in Table 1; 325 were male, and 248 were female. The 95 participants (16.57%) belonged to the age group of 20–29, 135 participants (23.56%) belonged to the age group of 30–39, 156 patients (27.22%) belonged to the age group of 40–49, and 187 patients (32.63%) belonged to the age group of above 50 years. The 102 participants (17.80%) had no formal education, 169 patients (29.49%) had a school education, 219 patients (38.21%) had completed college education, and 83 respondents (14.48%) had graduated from university.

**TABLE 1:** Demographics.

Description	*n*	Percentage
Gender		
Male	325	56.71
Female	248	43.28
Marital status		
Married	364	63.52
Unmarried	209	36.47
Age		
20–29	95	16.57
30–39	135	23.56
40–49	156	27.22
≥ 50	187	32.63
Education		
No formal education	102	17.80
School	169	29.49
College	219	38.21
University	83	14.48
Total	573	100

### 3.2. Hospital Comparability and Telehealth Representativeness

To ensure that the chosen institutions mirror a similar telehealth setting, we performed a preliminary analysis of the organizational, technological, and administrative features of the five hospitals included in the study. Hospitals are all under the Punjab Primary and Secondary Healthcare Department, which implements similar policies on the subjects of digital health, EMR integration, and TS deployment in public sector hospitals. The provincial government offers uniform infrastructure, teleconsultation policies, and digital service policies that lead to uniform telehealth provision and equal technological preparedness. Additionally, a pilot study (*n* = 53) assessed the interhospital reliability of the telehealth scale and the comparability of patient exposure to telehealth platforms; the values of Cronbach’s alpha were above acceptable limits, indicating that TS experiences were consistent at the sites [44]. Access and utilization patterns of telehealth were not found to have any systematic differences. These characteristics support the representativeness of the chosen hospitals as well as the validity of the moderation analyses in H8 to H12.

### 3.3. Construct Development and Research Instruments

All items were derived from previous studies and measured using a 5‐point Likert scale (1 = Very Strongly Disagree, 5 = Very Strongly Agree). Specifically, the 4‐item DHA measure was adapted from Abdulrahman et al. [45] and Kanwel et al. [5]. The 15‐item service quality in public hospital (SQPH) measure was sourced from Kanwel et al. [5] and Fatima et al. [46], whereas the four‐item DPC scale was obtained from Gao et al. [15]. PS was assessed using a nine‐item scale by Hussain et al. [47]. Finally, the TS scale, developed by Andrews et al. [28], comprises five items. Detailed items are provided in Appendix A.

### 3.4. Pilot Study

Our preliminary study helped us check the effectiveness of the questionnaire before starting primary research. A research group of 53 participants were selected from the total population, representing 7.06% of it. We tested the interrelatedness of the research items using IBM SPSS v.26 Statistics to calculate the Cronbach alpha. Researchers recognize a Cronbach alpha value of above 0.7 as sufficient measurement reliability [48]. Globally, the scientific community uses this standard when studying social science data. Our data indicate that the study constructs are highly reliable, meeting this criterion and demonstrating participant clarity in understanding the survey items [49].

### 3.5. Analysis Methods

To test the study hypotheses, a two‐step structural equation modeling (SEM) procedure, as suggested by Anderson and Gerbing [50], was used. We evaluated the measurement model using confirmatory factor analysis (CFA) in the first step and tested the structural model in the second step. IBM SPSS Amos 25.0 was used to perform maximum‐likelihood estimation as it is suitable for normally distributed data and is commonly applied in the field of SEM studies [51]. Cronbach’s alpha and composite reliability (CR) were used to assess the reliability of each construct, and both were above the recommended value of 0.70, indicating a high level of internal consistency [52]. Standardized factor loadings (> 0.50) and average variance extracted (AVE > 0.50) were used to evaluate convergent validity, whereas discriminant validity was evaluated using the Fornell–Larcker criterion. Several fit indices were applied to assess model fitness. The assessment was done based on the following cutoff criteria: *χ* ^2^/*d* *f* < 3, CFI, TLI, IFI ≥ 0.90, SRMR < 0.08, RMSEA < 0.06–0.08 [53, 54].

Descriptive statistics and correlations were calculated to investigate the preliminary relationships between the variables. Moreover, the Hayes PROCESS macro Version 3.1 was used to test the moderating effects of TSs, and 5000 bootstrapped samples were used to produce bias‐corrected confidence intervals [55]. This method allowed us to test the conditional effects at low, middle, and high levels of the moderator. This methodological design provides a rigorous assessment of the measurement and high testing of direct, indirect, and moderating variables based on the conceptual framework.

### 3.6. Measurement Model Validation

To make the measurement model more comprehensible to readers who might not be conversant with the concept of SEM, the measurement model was evaluated using the two‐step method suggested by Anderson and Gerbing [50], which requires an initial confirmation of the reliability and validity of the constructs, followed by the testing of structural relations. Internal consistency reliability was tested using Cronbach’s alpha and CR, both of which were higher than the suggested level of 0.70 [51]. Cronbach’s alpha and CR were evaluated, and CR is a more suitable measure in this research since it takes the standardized loading of the factors through CFA and does not presume that all the indicators have equal loading as Cronbach’s alpha. The fact that our constructs were represented reflectively in an SEM framework provides CR with a more precise estimate of internal consistency [51]. Hence, CR values have been taken as the major measure of construct reliability, whereas *α* values are provided as the reference. Convergent and discriminant validities were established based on standardized factor loading (> 0.50), average variance extracted (AVE 0.50), and the Fornell–Larcker criterion. This supplementary information creates transparency and enables readers to more effectively assess the strength of the measurement model.

The Cronbach alpha coefficient was used to evaluate the reliability of each construct. As shown in Table 2, all factor values exceeded the recommended cutoff threshold of 0.7 [48, 56]. Reference scales were established, and the measurement model was assessed using the CFA. The accepted factor loadings were above the 0.5 thresholds recommended in Ref. [51]. The measurement model fit indices (*χ* ^2^/*d* *f* = 2.265, NFI = 0.918, GFI = 0.942, SRMR = 0.036, RMSEA = 0.037) indicated an acceptable fit [53]. To assess internal consistency, both Cronbach’s alpha and CR were employed, with statistically significant results reported in Table 2 [44]. Convergent validity was established, with factor loadings ranging from 0.757 to 0.967, all exceeding the recommended value of 0.50 [52]. Discriminant validity was assessed using the square root of the AVE. Table 3 demonstrates the satisfactory discriminant validity as the square root of each construct’s AVE was more significant than the correlation between the latent variable pairs.

**TABLE 2:** Confirmatory factor analysis.

Construct	Item	Factor loading	*α*	AVE	CR
Digital health adoption	DHA1	0.795	0.855	0.679	0.952
	DHA2	0.847			
	DHA3	0.862			
	DHA4	0.766			

Service quality in public hospitals	SQPH1	0.874	0.847	0.767	0.962
	SQPH2	0.862			
	SQPH3	0.914			
	SQPH4	0.925			
	SQPH5	0.911			
	SQPH6	0.893			
	SQPH7	0.845			
	SQPH8	0.766			
	SQPH9	0.791			
	SQPH10	0.790			
	SQPH11	0.856			
	SQPH12	0.923			
	SQPH13	0.890			
	SQPH14	0.902			
	SQPH15	0.785			

Doctor–patient communication	DPC1	0.858	0.893	0.743	0.898
	DPC2	0.868			
	DPC3	0.757			
	DPC4	0.967			

Patient satisfaction	PS1	0.842	0.934	0.788	0.932
	PS2	0.887			
	PS3	0.864			
	PS4	0.809			
	PS5	0.810			
	PS6	0.943			
	PS7	0.788			
	PS8	0792			
	PS9	0.784			

Telehealth service	TS1	0.865	0.879	0.735	0.899
	TS2	0.877			
	TS3	0.783			
	TS4	0.919			
	TS5	0.790			

*Note: α* = Cronbach’s alpha.

Abbreviations: AVE, average variance extracted; DHA, digital healthcare adoption; DPC, doctor–patient communication; PS, patient satisfaction; SQPHs, service quality in public hospitals; TSs, telehealth services.

**TABLE 3:** Correlation and descriptive statistics.

Variables	Correlation of constructs					M	SD
	1	2	3	4	5		
1. DHA	**(0.824)**					3.875	1.252
2. SQPH	0.452^∗∗∗^	**(0.875)**				4.754	1.370
3. DPC	0.426^∗∗∗^	0.438^∗∗∗^	**(0.816)**			3.461	1.275
4. PS	0.486^∗∗∗^	0.462^∗∗∗^	0.511^∗∗∗^	**(0.887)**		4.112	1.201
5. TS	0.541^∗∗∗^	0.401^∗∗∗^	0.433^∗∗∗^	0.441^∗∗∗^	**(0.857)**	4.423	1.246

*Note:* M = Mean. Significance of correlations: ^∗^ *p* ≤ 0.05, ^∗∗^ *p* ≤ 0.01, ^∗∗∗^ *p* ≤ 0.001. The bold values on the diagonal represent the square root of the AVE.

Abbreviations: DHA, digital healthcare adoption; DPC, doctor–patient communication; PS, patient satisfaction; SD, standard deviation; SQPHs, service quality in public hospitals; TSs, telehealth services.

### 3.7. Structural Model

**FIGURE 2:**
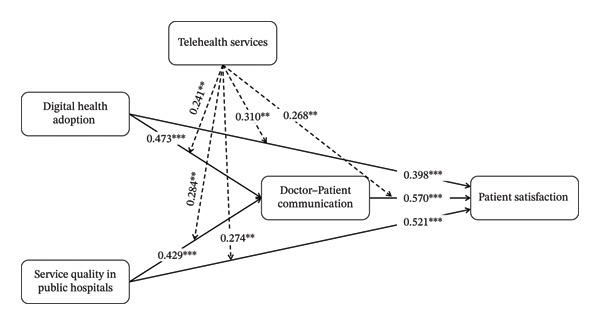
Results of structural equation modeling. Note: ^∗^ *p* < 0.05; ^∗∗^ *p* < 0.01; ^∗∗∗^ *p* < 0.001.

**FIGURE 3:**
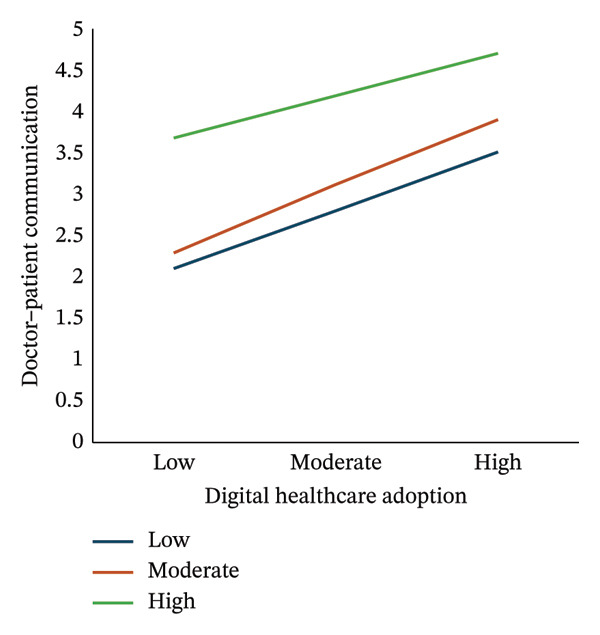
TS moderation between DHA and DPC.

**FIGURE 4:**
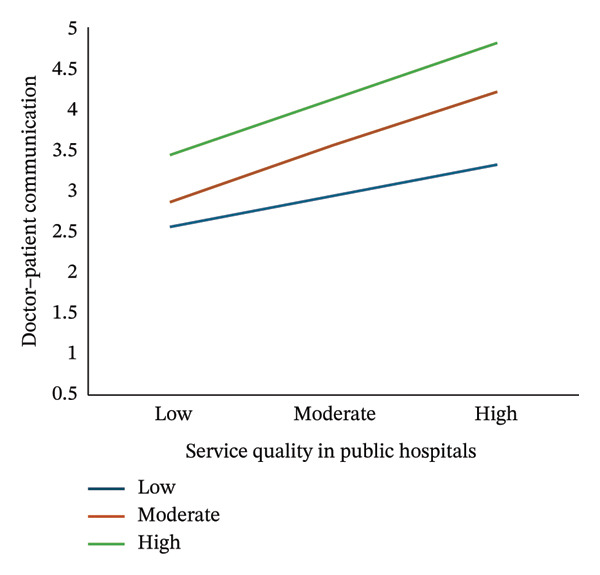
TS moderation between SQPH and DPC.

**FIGURE 5:**
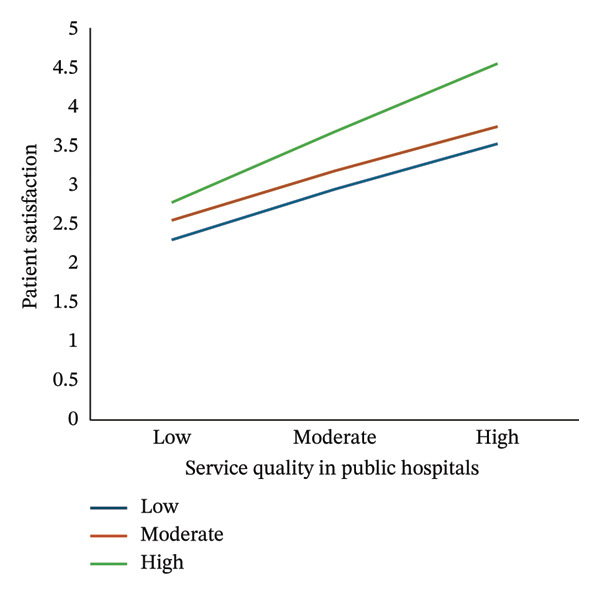
TS moderation between SQPH and PS.

**FIGURE 6:**
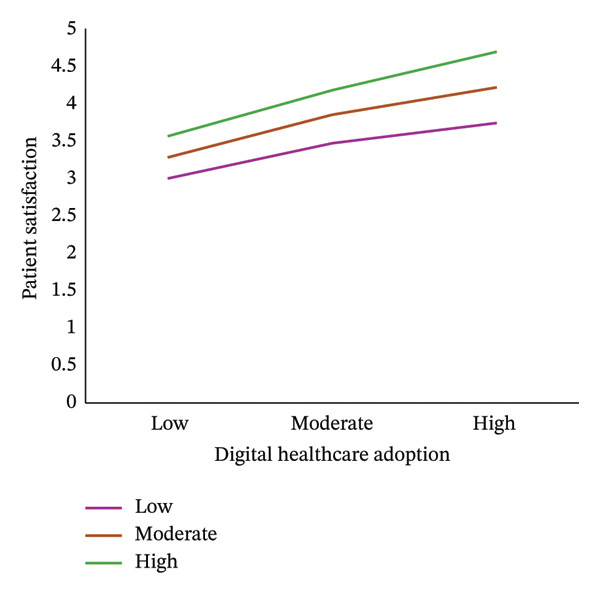
TS moderation between DHA and PS.

**FIGURE 7:**
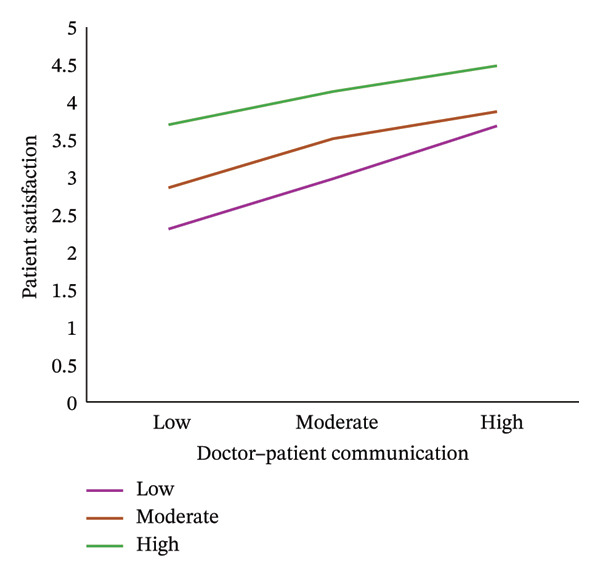
TS moderation between DPC and PS.

The outcomes of SEM are revealing that there is a good model fit (*χ* ^2^/*d* *f* = 2.247, CFI = 0.935, NFI = 0.925, TLI = 0.913, IFI = 0.931, RMSEA = 0.065, SRMR = 0.039). Table 4 presents the direct and indirect path coefficients. Use of digital healthcare is a strong predictor of DPC (*β* = 0.473, *t* = 8.298, *p* ≤ 0.01), supported H1. SQPH also shows a strong positive influence on DPC (*β* = 0.429, *t* = 8.755, *p* ≤ 0.01), which validates H2. DHA has a positive impact on PS (*β* = 0.398, *t* = 7.803, *p* ≤ 0.01), which supports H3, whereas SQPH has various effects on PS (*β* = 0.521, *t* = 6.946, *p* ≤ 0.01), which supports H4. Moreover, H5 is supported because DPC is a significant predictor of PS (*β* = 0.570, *t* = 8.028, *p* ≤ 0.01). Bootstrapped indirect effects proved the mediating effect of DPC in both DHA ⟶ PS (95% CI = 0.141, 0.283) and SQPH ⟶ PS (95% CI = 0.259, 0.375) relationships, supporting H6 and H7. Moderation results (Table 5) indicate that TSs moderate all the five relationships tested significantly: DHA ⟶ DPC (*β* = 0.241, *t* = 3.459, *p* ≤ 0.011), SQPH ⟶ DPC (*β* = 0.284, *t* = 3.251, *p* ≤ 0.015), SQPH ⟶ PS (*β* = 0.274, *t* = 3.206, *p* ≤ 0.0017), DHA ⟶ PS (*β* = 0.310, *t* = 3.968, *p* ≤ 0.011), DPC ⟶ PS (*β* = 0.268, *t* = 3.891, *p* ≤ 0.019). Figures 2, 3, 4, 5, 6, and 7 indicate these interaction effects, which show more significant interaction effects at increased levels of TSs. In general, all assumed direct, indirect, and moderating relationships were confirmed by the results of the formulated conceptual model.

**TABLE 4:** Direct and indirect *β* coefficients.

Hypotheses	Path	*β*	SE	*t* value	*p* value	Bias‐corrected 95% CI	
						LLCI	ULCI
*Direct effects*							
H1	DHA ⟶ DPC	0.473	0.057	8.298	0.001	0.392	0.478
H2	SQPH ⟶ DPC	0.429	0.049	8.755	0.001	0.387	0.512
H3	DHA ⟶ PS	0.398	0.051	7.803	0.001	0.238	0.462
H4	SQPH ⟶ PS	0.521	0.075	6.946	0.001	0.379	0.543
H5	DPC ⟶ PS	0.570	0.071	8.028	0.001	0.281	0.398

*Indirect effects*							
H6	DHA ⟶ DPC ⟶ PS	0.197	0.062	3.177	0.001	0.141	0.283
H7	SQPH ⟶ DPC ⟶ PS	0.189	0.059	3.203	0.001	0.259	0.375

Total effect		0.386	0.121	6.380	0.001	0.230	0.382

*Note:* Significance: ^∗^ *p* ≤ 0.05, ^∗∗^ *p* ≤ 0.01, ^∗∗∗^ *p* ≤ 0.001.

Abbreviations: DHA, digital healthcare adoption; DPC, doctor–patient communication; LLCI, lower‐level confidence interval; PS, patient satisfaction; SQPHs, service quality in public hospitals; TSs, telehealth services; ULCI, upper‐level confidence interval.

**TABLE 5:** Moderating effect test.

Paths	*β*	SE	*t* value	*p* value	Bootstrapping	
					LLCI	ULCI
Moderation Model 1 (dependent DPC)						
DHA	0.6232	0.0644	9.6770	0.0000	0.4701	0.6575
TS	0.5483	0.0689	7.9579	0.0000	0.3524	0.6215
DHA × TS	0.2411	0.0697	3.4591	0.0011	0.2175	0.3712
Conditional effects						
Low	0.6462	0.0785	8.2318	0.0000	0.3667	0.5404
Medium	0.3523	0.0528	6.6723	0.0000	0.3489	0.4891
High	0.3641	0.0746	4.8806	0.0000	0.2915	0.3228
Moderation Model 2 (dependent DPC)						
SQPH	0.6574	0.0785	8.3745	*p* ≤ 0.001	0.3251	0.5267
TS	0.5832	0.0832	7.0096	*p* ≤ 0.001	0.5231	0.6163
SQPH × TS	0.2845	0.0875	3.2514	*p* ≤ 0.01	0.4592	0.6933
Conditional effects						
Low	0.7453	0.0721	10.3370	*p* ≤ 0.001	0.3275	0.5903
Medium	0.4562	0.0545	8.3706	*p* ≤ 0.001	0.3491	0.5572
High	0.3254	0.0697	4.6685	*p* ≤ 0.001	0.2579	0.6243
Moderation Model 3 (dependent PS)						
SQPH	0.5748	0.0736	7.8097	*p* ≤ 0.001	0.4589	0.5387
TS	0.4279	0.0759	5.6376	*p* ≤ 0.001	0.6972	0.7934
SQPH × TS	0.2745	0.0856	3.2067	*p* ≤ 0.01	0.2180	0.3266
Conditional effects						
Low	0.5671	0.0526	10.7813	*p* ≤ 0.001	0.4577	0.5626
Medium	0.3724	0.0491	7.5845	*p* ≤ 0.001	0.5114	0.8059
High	0.2376	0.0662	3.5891	*p* ≤ 0.05	0.3671	0.5190
Moderation Model 4 (dependent PS)						
DHA	0.8967	0.1082	8.2874	*p* ≤ 0.001	0.8934	1.4314
TS	0.5661	0.0873	6.4845	*p* ≤ 0.001	0.4598	0.9290
DHA × TS	0.3107	0.0783	3.9680	*p* ≤ 0.05	0.6712	0.8775
Conditional effects						
Low	0.6317	0.0518	12.1949	*p* ≤ 0.001	0.3865	0.5278
Medium	0.4376	0.0582	7.5189	*p* ≤ 0.001	0.4132	0.5845
High	0.2456	0.0548	4.4817	*p* ≤ 0.05	0.3774	0.5945
Moderation Model 5 (dependent PS)						
DPC	0.7267	0.0775	9.3767	*p* ≤ 0.001	0.2965	0.4367
TS	0.3231	0.0529	6.1077	*p* ≤ 0.001	0.2451	0.3658
DPC × TS	0.2689	0.0691	3.8914	*p* ≤ 0.01	0.4230	0.6068
Conditional effects						
Low	0.4271	0.0688	6.3937	*p* ≤ 0.01	0.3665	0.4592
Medium	0.2459	0.0563	4.3676	*p* ≤ 0.01	0.2390	0.5342
High	0.3282	0.0843	3.8932	*p* ≤ 0.05	0.2076	0.2938

*Note:* Significance: ^∗^ *p* ≤ 0.05, ^∗∗^ *p* ≤ 0.01, ^∗∗∗^ *p* ≤ 0.001.

Abbreviations: DHA, digital healthcare adoption; DPC, doctor–patient communication; LLCI, lower‐level confidence interval; PS, patient satisfaction; SQPHs, service quality in public hospitals; TSs, telehealth services; ULCI, upper‐level confidence interval.

The moderation hypotheses (H8, H9, H10, H11, and H11) were tested using Hayes′ PROCESS macro 3.1 [55]. The results presented in Table 5 and Figure 3 confirm H8, indicating that (TSs moderate the relationship between DHA and DPC (*β* = 0.241, *t* = 3.459, *p* ≤ 0.0011). Additionally, H9 is supported, indicating that SQPH and DPC have a positive moderating effect through TS (*β* = 0.284, *t* = 3.251, *p* ≤ 0.0015) (Figure 4). Furthermore, the findings show a significant TS relationship between SQPH and PS (*β* = 0.274, *t* = 3.206, *p* ≤ 0.0017), thereby supporting H10 (Figure 5). The association between DHA and PS is moderated by TSs (*β* = 0.310, *t* = 3.968, *p* ≤ 0.0011), supporting H11 (Figure 6). TS also positively moderates between DPC and PS with (*β* = 0.268, *t* = 3.891, and *p* ≤ 0.0019), supporting H12 (Figure 7). Overall, TS interactions play a significant role in these relationships. The moderating effects of H8, H9, H10, H11, and H12 are illustrated in Figures 2, 3, 4, 5, 6, and 7.

## 4. Discussion

The DHA dimensions of EHRs, DPS, and technology sophistication enhance doctor–patient communication through better information access, reduced paperwork, and seamless connections between staff. The storage and retrieval processes in EHRs facilitate the maintenance of clinical continuity across treatments and improve data‐based treatment decisions [57]. According to Agarwal et al. [58], DPS speeds up transactions, enabling doctors to dedicate more time to interacting with patients than completing administrative work. Advanced medical technologies, including telemedicine‐ and AI‐driven chatbots, together with patient portals, provide patients with various communication options to obtain prompt and customized healthcare services beyond in‐person doctor visits [59]. Digital integration establishes trust while boosting PS and strengthening relationships with doctors because it provides healthcare communication that is clear and accessible through responsive services [60].

Although the data in this study offer solid evidence based on five large state‐owned hospitals in Punjab, the overall applicability of these findings remains questionable. The administrative and digital health governance structure in Punjab is relatively uniform; thus, it is an analytically homogenous setting. However, provincial heterogeneity in Pakistan, such as disparities in infrastructural investments, digital literacy, and human resource accessibility, can change the intensity of relationships found in this setting. For example, areas such as Balochistan or interior Sindh have a higher degree of connectivity restrictions and resource scarcity, which may affect both DHA and PS rates. Outside Pakistan, the results can be conceptually generalized to other countries with lower‐ and middle‐level incomes (e.g., India, Bangladesh, Nigeria, and Kenya), where the digital transformation of the public healthcare sector is underway and has similar structural limitations. However, cross‐regional comparative studies are required to confirm whether telehealth moderates service quality and PS similarly across various healthcare systems.

The public health dimensions of service quality, consisting of doctor services, nurse services, pharmacy services, and laboratory services, facilitate improved doctor–patient communication by creating cooperative and efficient healthcare settings. Excellent doctor service standards provide doctors with sufficient resources, patient details, and ample time to conduct meaningful discussions that result in accurate medical information and improved patient–doctor decision‐sharing practices [61]. Nurse services enhance doctor–patient communication through continuous support, relevant patient education, and emotional comfort, which overcomes the gaps in doctor‐to‐patient relationships [62]. Pharmacy services support communication efforts by providing detailed medication guidance and adherence instructions, which reduce mistakes and health risks [63]. Laboratory services enhance diagnostic reliability and streamlines reporting processes, enabling physicians to deliver specific evidence‐based medical consultations to patients, leading to improved patient cooperation in their healthcare [26]. The combined service quality dimensions form a patient‐focused system that enhances DPC by delivering clarity and trust and improving health outcomes.

Patients experienced increased satisfaction with the DHA dimensions, including EHRs, DPS, and technology sophistication. Medical efficiency increases through EMR implementation by preventing errors, enhancing diagnostics, and delivering better interactions between healthcare professionals, resulting in superior patient outcomes and quality of care [57]. DPS enables faster billing procedures, reduces administrative work, and enhances patient convenience and satisfaction [58]. Patients receive better treatments through healthcare advancements, such as telehealth and diagnostic AI, which advance personalized care, followed by early medical treatments, thus building trust [59].

Although DHA and telehealth positively influence healthcare, cybersecurity, ineffective digital infrastructure, and unreliable internet access limit the benefits of digital care, particularly in rural Pakistan. Enhancing cyber‐health standards at the national level, investing in the secure architecture of EHRs, and broadband coverage are critical steps toward maintaining patient trust and the uptake of digital services [4, 17]. The inclusion of state‐sponsored programs on digital literacy would reduce system abuse and strengthen users’ trust in remote care solutions.

Technology sophistication in healthcare settings enhances operational efficiency and service quality, thereby improving PS. Additionally, hospitals with more digital infrastructure, such as organizations with systems that perform automated appointment scheduling and AI decision‐support systems, can provide services to their patients faster and more accurately, reducing frustration and improving the general experience of patients [12]. Furthermore, digital innovations have been shown to enhance access to healthcare, particularly for patients in remote areas, thereby ensuring timely medical consultations and appointments. In this case, as patients demand digital solutions in healthcare, effective implementation of EHRs, DPS, and advanced technologies will encourage a patient‐centered approach to deliver higher satisfaction and loyalty from patients [19].

Enhancing the provision and delivery of publicly funded healthcare in hospitals, including doctor services, nurse services, and other specialized areas, strengthens PS. Doctor service quality that includes the correct diagnoses and personal attention helps patients trust their providers and enhances satisfaction, according to Lee et al. [61]. According to Asif et al. [40], nurse services offer quality care and quick help alongside emotional support to ensure that patients feel safe and at ease within the healthcare system, according to Asif et al. [40]. Through proper medication delivery, pharmacy services help patients better understand their healthcare plans, while reducing medication concerns. According to Kharaba et al. [36], laboratory services provide excellent care because it promptly produces accurate test results, enabling doctors to promptly initiate treatment. These service quality dimensions form a patient‐friendly care model that fosters trust and reduces anxiety, ultimately improving the healthcare for patients who express higher levels of satisfaction.

Doctors’ communication skills help bridge the relationship between DHA components, EHRs, and DPS, as well as technology sophistication and PS, by creating an environment in which information flows smoothly, trust builds, and services improve. EHRs facilitate effortless patient record access, leading to accurate diagnoses and enhanced consultation quality, which, in turn, fosters strong interactions between doctors and patients [19]. DPS streamlines administrative work, allowing doctors to dedicate more time to direct patient care and provide an enhanced healthcare experience [12]. The implementation of sophisticated technological features, such as telehealth and AI support systems, makes care more accessible and responsive. Hence, patients receive prompt healthcare advice and ongoing engagement, leading to improved satisfaction [15]. The effective integration of digital innovations enhances doctor–patient communication through transparent hospital practices, reduces errors, and fosters better patient involvement, resulting in improved healthcare outcomes [46].

DPC is the primary link between various aspects of service quality and PS through thorough and open healthcare interactions. Quality doctor services improve medicine through clear discussions and patient involvement in treatment decisions, which produce more satisfied patients [26]. Nurse services members establish better doctor–patient links through ongoing care discussions and ensure that patients receive teaching materials from their medical support team [62]. Pharmacy service members explain medications well and teach patients how to take their medicines, leading to a more straightforward understanding and better results in treatment [12]. Through diagnostic accuracy, laboratory services help doctors and patients share information during discussions [9]. The connected work of these service quality elements in DPC helps both parties understand each other better, calms patients, and makes them pleased with their healthcare experiences.

### 4.1. TSs as a Moderator

The incorporation of TSs serves as a fundamental moderating link to enhance the correlation between DHA dimensions and DPC through improved accessibility, process efficiency, and increased patient engagement. Doctors can smoothly access clinical histories through combined telehealth and EMR systems to deliver personalized and knowledgeable consultations to patients in underserved areas [57]. According to Agarwal et al. [58], DPS within TSs provides secure payment processing capabilities that eliminate time‐consuming administrative tasks and enable medical staff to dedicate their efforts to patient interaction. System‐enabled diagnostic capabilities, together with virtual consulting tools through AI, enhance doctor–patient interaction capabilities by providing instant clinical support and reaching patients across various geographic settings [59]. TSs, which maintain uninterrupted communication between healthcare providers and patients, reinforce the impact of DHA.

The implementation of TSs has a moderating effect on the relationship between service quality and DPC, as it enables more efficient, patient‐focused healthcare meetings. Virtual consultations enable doctors to establish reliable follow‐up support alongside immediate medical consultations, which builds strong doctor–patient trust relationships [19]. Nurses implement telehealth systems to deliver distance‐based patient educational services combined with health status monitoring and emotional support, which keeps treatment plans active in their care [61]. The remote counseling capabilities of tele‐pharmacy services enable pharmacists to deliver instructions about medication adherence and potential side effects to patients, thereby preventing health hazards caused by miscommunication [18]. Remote laboratory reporting systems enable virtual patient access to test outcomes and result discussions, thereby enhancing transparency and facilitating quick medical decisions [16]. TSs strengthen the service quality dimensions of healthcare by enabling better patient access to efficient communication that focuses on their specific needs.

Telehealth creates a bridge between modern technology and healthcare services and improves physician‐to‐patient interactions, healthcare services, and consumer satisfaction. Telehealth, combined with the DHA and service quality dimensions, creates better medical consultation efficiency, together with improved trust and reduced patient stress, which develop both a responsive and interactive medical environment [5]. Digital healthcare transformation requires TSs to fulfill their moderating function, enabling healthcare providers to achieve effective doctor–patient exchanges and enhance both patient health outcomes and engagement.

Healthcare services act as a moderating variable in improving DHA dimensions, including EHRs, DPS, and technology sophistication, by ensuring that online service delivery increases for some patients, as well as enhancing the efficiency and convenience of service delivery. Integrating telehealth with EHRs makes data sharing efficient and provides real‐time access to patient documents, which may decrease multiple processes and enhance patient experience, which is beneficial for patients [34]. Consequently, the integration of DPS into telehealth platforms permits patients to enter into financial transactions, avoids long waiting times, and reduces administrative costs, thus offering a better client experience in receiving care [17]. Furthermore, technological advancements, particularly the use of AI diagnostics and remote monitoring of patients, increase healthcare access and corrective responses to ensure timely attention and engagement, which not only improves satisfaction with healthcare services [4]. Therefore, TSs build on the achievements of DHA by augmenting the utilization factor, reducing patients’ inconvenience, and enhancing patient‐centered outcomes.

The introduction of TSs helps connect DHA with service quality, enabling better PS through improved care delivery and enhanced interactions between patients and healthcare providers. Doctor services benefit from virtual consultations because doctors can provide patient care anywhere, while decreasing travel challenges and making professional healthcare easier to reach [19]. Nurses use telehealth technologies to help patients through online support calls, while teaching them about their care needs and monitoring their condition after discharge, which builds trust in medical treatments [62]. Through tele‐pharmacy services, patients gain better satisfaction by receiving quick medication support and prescription advice, improving treatment success, and addressing medicine‐related questions [63]. Remote laboratory services enable healthcare providers to deliver test results electronically and engage in online discussions with patients. According to Lee et al. [61], this rapid diagnostic exchange makes patients and others feel better according to Lee et al. [61]. Telehealth tools develop and perfect healthcare service quality dimensions to create user‐friendly and convenient services.

Telehealth combinations of new technology and quality services help patients become more satisfied with their healthcare experiences. Integrating telehealth with the DHA and service quality dimensions creates better healthcare service delivery results, lowers patient anxiety, and makes them more comfortable with their providers [61]. The expanding digital healthcare world requires TSs to deliver better care to patients in ways that bring them greater comfort and faster outcomes.

### 4.2. Contribution to the Literature

This work contributes to the existing body of the literature in three ways, directly filling the gaps in the saturated digital health literature. First, it provides empirical data of Pakistani general hospitals with low digital maturity, insufficient EMR uptake, disjointed policies, and limited resources, and where the TAM has seldom been operationalized or validated [2–4]. This is in contrast to the prevailing literature on digitally mature, privatized, or consumer‐driven health systems [1, 16]. Second, the study expanded the existing research by incorporating four of the key constructs of public sector service quality (doctor, nurse, pharmacy, and laboratory services) in the technology–satisfaction pathway, as researchers have acknowledged that digital technologies can only impact patient outcomes when they are implemented instead of core clinical and operational processes [13, 15, 46]. These structural integration areas have not been well studied in the literature, as studies have usually focused on service quality or technology adoption separately. Third, this study presents a multipath moderation framework in which telehealth alters five different relationships among digital adoption, service quality, communication, and satisfaction. Earlier studies have tended to evaluate the moderating effect of telehealth as a direct antecedent or single‐path agent; however, little information is available on its systemic moderating impact as a component of complex healthcare delivery models [17–19].

## 5. Conclusion

In the Pakistani healthcare sector, the establishment of EHRs, DPS, and sophisticated TSs has the potential to bring significant changes. Despite all the concerns related to inequity that exist in the population of developing countries in terms of social and economic status, digital divide, weak infrastructure, and many more challenges, the integration of such technologies can increase the quality of service delivery and improve communication between doctors and patients, which may also lead to an increase in overall satisfaction among patients. TSs have tremendous potential to address the issue of geographical distance and shortage of physicians and other healthcare providers, particularly those located in rural and underserved regions, by offering remote consultations, prescription services, and continuous medical check‐ups. However, these digital interventions cannot be fully implemented because of challenges such as internet connectivity issues, cybercrimes, and the reluctance of healthcare professionals. This means that leaders in healthcare settings and politicians need to invest in appropriate structures and technology as well as in adequate guidelines, best practices, and education programs that will help users of these technologies.

A laboratory examination of PS preferences in Pakistan aligns with global digital health studies, which show that healthcare efficiency is enhanced by improved accessibility and service quality. The United States, Germany, and the United Kingdom are examples of developed nations that have successfully implemented digital healthcare solutions. Studies have demonstrated the need for patient‐focused strategies that integrate digital technology platforms with high‐quality healthcare services to foster open communication between patients and healthcare providers. TSs have played a crucial role in enhancing the relationship between DHA, service quality, and PS in current remote healthcare delivery systems following the COVID‐19 pandemic.

To make digital healthcare and telehealth viable in the long term in Pakistan, policymakers should focus on cybersecurity standards, reliable connectivity, and capacity building for technology implementation in public hospitals. These systemic barriers should be addressed to reduce digital risks and enhance the moderating role of telehealth in PS. Additionally, the situation in Punjab is just one part of the overall picture of the health system in Pakistan, and new comparative research among provinces might help better researchers, policymakers, and stakeholders understand how digital preparedness, governmental competence, and infrastructural inequality condition the generalizability of these findings. This cross‐regional evidence offers a ground‐breaking basis for national‐level digital health policy development.

The combination of digital healthcare innovations, service quality improvements, and telehealth mediation has excellent potential to enhance public healthcare satisfaction in Pakistan. This study establishes a comprehensive foundation to support further research and policy efforts, resulting in fair and efficient healthcare service delivery across Pakistan and globally.

### 5.1. Theoretical Contributions

This study has several theoretical implications for digital healthcare, technology acceptance, and nursing management. First, it contributes to the development of the TAM by integrating the essential dimensions of service quality, such as doctor services, nurse services, pharmacy services, and laboratory services into the behavioral pathway between technology adoption and PS. Although the TAM has been extensively used in technologically developed settings, little research has been conducted using it in low‐digital‐maturity–structured public health systems. Our results indicate that the acceptability of technology does not provide total responsibility for patient‐centered outcomes without involving basic service delivery procedures that nurses and other related healthcare professionals directly impact. This healthcare‐specific extension of the TAM is context‐based and offers a more service‐focused and relational context for clinical systems.

Second, the research provides new empirical findings by establishing a multipath moderating effect of TSs in five structural models (DHA ⟶ DPC, SQ ⟶ DPC, DHA ⟶ PS, SQ ⟶ PS, DPC ⟶ PS). The fact that telehealth is often viewed as a predictor of satisfaction or access indicates that its effects as a structural amplifier of technology–service–outcome pathways are poorly understood. Our findings indicate that telehealth does not replace face‐to‐face interactions; rather, it complements the mechanisms involved in translating digital uptake and service quality into improved communication and satisfaction. This observation contributes to the theoretical knowledge that telehealth is an integrative digital modulation that transforms the behavioral and experiential aspects of care.

Third, the analysis addresses the international demands of nursing management research by responding to calls to better theorize on the intersection of digital infrastructure, provider communication, and patient‐centered outcomes. Digital transformation strategies in most Organisation for Economic Co‐operation and Development (OECD) and Asian healthcare processes focus on hybrid care models, interoperability, clinical communication platforms, and nurse‐led teleconsultation services. Placing the Pakistani context in this international path reveals that, despite resource‐limited environments, the digital adoption process can improve service quality and communication, which are some of the fundamental constructs of nursing management theory, when telehealth tools are implemented as an enabling process and not as a single innovation. Taken together, these contributions provide an integrated, context‐specific theoretical framework for perceiving technology‐mediated PS, especially for governments with public health systems in the early stages of digital transformation.

### 5.2. Practical Contributions

Healthcare administrators, nursing leaders, digital health planners, and policymakers can learn valuable practical lessons from this study. First, the findings emphasize that digital healthcare implementation can bring tangible communication and satisfaction outcomes only when there is a high level of clinical service quality. To the nursing managers, this highlights the strategic effect of matching digital efforts, including EMR integration and DPS, as well as AI‐powered clinical tools, with workforce abilities, patient‐flow redesign, and communication training. Digital transformation will not exist exclusively in the form of a technological process and will have to be integrated into nursing processes, interprofessional collaboration, and quality‐of‐care standards.

Second, the results underline the pivotal importance of telehealth as a strategic accelerator for enhancing patient engagement, continuity of care, and communication transparency. The moderating impact of telehealth policy investments should focus on the stable connectivity and teleconsultation education of nurses and physicians as well as on standardized teletriage guidelines. These improvements can minimize unnecessary travel strain, expand access to rural and underserved regions, and aid in case management efforts usually carried out by nurses. In LMIC health systems, where the shortage of specialists and geographic inequities are severe, telehealth‐facilitated hybrid care is a viable and extensively extendable path toward patient experience.

Third, the study reveals the necessity of combining digital governance models with the leadership of hospitals and national regulatory agencies. The achievement of patient‐centered benefits can be hindered by fragmented or inconsistent EHRs and telehealth platform implementation. Having interoperable systems, a common digital documentation standard, and effective data governance policies would make patient information exchanges more reliable and improve communication quality.

Finally, the findings will inform stakeholders that investments in nurse training, digital literacy initiatives, communicative competencies, and user‐friendly digital interfaces are not merely professional upgrades but also essential PS outcomes. With global healthcare systems shifting toward digital maturity models that focus on workforce preparedness and patient engagement, this research provides a viable approach to adjust the models to the conditions of limited resources in state‐provided healthcare.

### 5.3. Limitations and Transferability

Although this study presents important empirical data regarding the effects of digital healthcare uptake, service quality, and telehealth on PS, there are several limitations that should be considered when interpreting the results and conducting future research. First, the research was carried out in only five large hospitals in the public sector in Punjab Province, which, although centrally managed and somewhat consistent in the digital implementation of health policies, does not reflect the wider structural and digital preparedness gap in Pakistan. Other provinces, such as Balochistan, Khyber Pakhtunkhwa, and Sindh, stand out significantly in terms of ICT infrastructure, broadband uptake, integration of public and privately provided health services, and digital literacy of their workforce. This can lead to significant differences in the strength and direction of relationships, especially the moderating effects of telehealth, in situations with less connectivity, less coherent health governance, or fewer digital competencies among frontline nursing personnel.

Second, the cross‐sectional design does not allow causal conclusions to be drawn. Healthcare digital transformation is dynamic, and variables, including the normalization of telehealth, patient trust, and organizational digital maturity, may change quickly. Longitudinal or experimental designs would be more appropriate for monitoring changes in behavioral acceptance and patterns of communication as health systems continue to become increasingly digitalized.

Third, the study was based on self‐reported perceptions of service quality, telehealth experience, and satisfaction, which are prone to common method bias and context‐dependent interpretations. Although sound measurements and reliability methods were used, future research may use multisource data, such as clinical outcomes, digital utilization records, patient–provider interaction time, and nursing workflow metrics.

Fourth, the results need to be cautiously extended to LMICs other than Pakistan. Although some LMICs have common challenges, such as a lack of infrastructure, personnel gaps, and inter‐regional heterogeneity of digital maturity, there are still significant differences between regions. For example, African and Southeast Asian countries with developed digital governance systems (e.g., Singapore and Malaysia) may react differently to telehealth‐mediated care pathways. Therefore, a cross‐country comparative study would enhance the knowledge of how governance capacity, digital norms, and regulatory environments determine the adoption of the satisfactory mechanisms that this study has determined. In general, this study’s limitations indicate that multilevel, multimethod, and cross‐regional research designs are required to validate and generalize the findings to other global health systems.

## Author Contributions

Conceptualization: Shahida Kanwel; methodology: Shahida Kanwel and Abid Hussain; validation: Zhiqiang Ma, Bailin Ge, and Mingxing Li; formal analysis: Shahida Kanwel; investigation: Shahida Kanwel, Mingxing Li, Abid Hussain, Arif Jameel, and Saif Ahmed; resources: Zhiqiang Ma, Saif Ahmed, and Bahar Hussain; data curation: Shahida Kanwel and Abid Hussain; writing–original draft preparation: Shahida Kanwel; writing–review and editing: Shahida Kanwel, Zhiqiang Ma, Mingxing Li, Abid Hussain, Arif Jameel, Bailin Ge, Bahar Hussain, and Bahar Hussain.

## Funding

This study was funded by the National Natural Science Foundation of China (Grant Nos.: 71573109 and 71974082) and the Jiangsu Funding Program for Excellent Postdoctoral Talent (Grant No.: 2024ZB890).

## Ethics Statement

Ethical approval for this study was obtained from the Institutional Human Research Ethics Committee (HREC) of the Islamia University of Bahawalpur. Approval was formally granted during a committee meeting held on June 12, 2024, as documented in an official approval letter (Ref. No. PA/112/2024, HREC/2024), who reviewed the research methodology, project summary, and ethical checklist. This study complies with the Ethical Principles of Psychologists and Code of Conduct of the American Psychological Association and the Declaration of Helsinki.

## Consent

Human volunteers participated in this study, which followed established ethical guidelines. Before participating, all participants were thoroughly advised about the aims of the research, methods, optional character of their participation, and the confidentiality of their responses. All participants provided written informed consent prior to questionnaire delivery. Because every participant was an adult capable of providing informed consent, proxy consent processes were not required. Informed consent was obtained during the data collection period from August 5, 2024, to December 27, 2024.

## Conflicts of Interest

The authors declare no conflicts of interest.

## Data Availability

Data supporting the findings of this study are available upon request from the corresponding author. The data are not publicly available because of privacy and ethical restrictions.

## Appendix A

### Appendix A1: Digital Health Adoption (DHA) [5, 45]

• DHA1: DHA is much more convenient and user‐friendly as compared to the paper‐based conventional payment system.
• DHA2: The digital payment system is quicker than the traditional payment system.
• DHA3: I trust the ability of digital payment systems to protect my privacy & Transactions.
• DHA4: I thought that it is not difficult to get the framework to do what I needed it to do.

### Appendix A2: Service Quality in Public Hospitals (SQPH) [5, 46]

• SQPH1: Received the test results in time.
• SQPH2: Procedures and tests explained well.
• SQPH3: I received an adequate explanation of any tests I had to undergo.
• SQPH4: The pharmacy staff seems to have a genuine interest in me as a person.
• SQPH5: If I have a question about my prescription, the pharmacist is always available to help me.
• SQPH6: The pharmacist is good at explaining things in a way that I understand.
• SQPH7: There was a pleasant atmosphere in the ward.
• SQPH8: The staff was professional.
• SQPH9: Toilet facilities were clean.
• SQPH10: Medical procedures were performed correctly the first time.
• SQPH11: The doctors seemed to understand how I experienced my situation.
• SQPH12: I have some doubts about the ability of the doctors who treat me.
• SQPH13: Services were provided efficiently.
• SQPH14: The nursing staff treated you as an individual.
• SQPH15: Rules and regulations were strictly maintained.

### Appendix A3: Doctor‐Patient Communication (DPC) [15]

• DPC1: Doctors are good about explaining the reason for medical tests.
• DPC2: The doctors were willing to answer any questions.
• DPC3: Sometimes doctors make me wonder if their diagnosis is correct.
• DPC4: Doctors sometimes ignore what I tell them.

### Appendix A4: Patient Satisfaction (PS) [47]

• PS1: The services of the healthcare provided by OPD are convenient.
• PS2: I find it hard to get an appointment for medical care right away.
• PS3: I can get medical care whenever I need it.
• PS4: I have easy access to the medical specialist I need.
• PS5: I feel confident that I can get the medical care I need without being set back financially.
• PS6: I have to pay for more of my medical care than I can afford.
• PS7: The medical care, I have been receiving is just about perfect.
• PS8: I think my doctor’s office has everything needed to provide complete care.
• PS9: When I go for medical care, they are careful to check everything when treating and examining me.

### Appendix A5: Telehealth Services (TS) [28]

• TS1: Telehealth provides greater access to healthcare specialists.
• TS2: Telehealth provides greater overall healthcare access.
• TS3: Telehealth provides safety from exposure to diseases.
• TS4: Telehealth provides excellent health care.
• TS5: Telehealth effectively improved my medical condition.
